# Brachytherapy for cervical cancer: from intracavitary to interstitial technique

**DOI:** 10.3389/fonc.2024.1442712

**Published:** 2024-11-06

**Authors:** Xiaojing Yang, Hanru Ren, Zhen Li, Jie Fu

**Affiliations:** ^1^ Department of Oncology, Shanghai Sixth People’s Hospital Affiliated to Shanghai Jiao Tong University School of Medicine, Shanghai, China; ^2^ Department of Orthopedics, Shanghai Pudong Hospital, Pudong Medical Center, Fudan University, Shanghai, China

**Keywords:** cervical cancer, brachytherapy, intracavitary brachytherapy, interstitial brachytherapy, radioactive seed implantation

## Abstract

Cervical cancer is a common malignant tumor of female reproductive system. Radiation therapy is one of the main methods of cervical cancer treatment, of which brachytherapy is an essential and important part of radiation therapy for locally advanced cervical cancer. With the rapid development of imaging technologies such as computed tomography (CT) and magnetic resonance imaging (MRI), brachytherapy for cervical cancer has gradually developed from traditional two-dimensional image-guided technology to three-dimensional image-guided technology. And there are more and more treatment methods, including intracavitary brachytherapy, interstitial brachytherapy, and intracavitary combined interstitial implantation brachytherapy. We performed a PubMed search for introduce the application progress of intracavity, implantation, intracavity combined implantation brachytherapy and radioactive seed implantation, and discuss the dosimetric feasibility of internal and external fusion irradiation.

## 1 Introduction

Cervical cancer is one of the most common malignant tumors in women (1). According to the 2020 Global Cancer Statistics, there were 604,127 new cervical cancer patient cases and 41,831 cervical cancer deaths that year (2). Generally speaking, patients with early cervical cancer have no obvious symptoms and signs, and most of the patients are in the middle and late stages when they are discovered. For locally advanced cervical cancer, radiotherapy is the main treatment method (3). The internationally recognized standard treatment for locally advanced cervical cancer is external irradiation + concurrent chemotherapy + brachytherapy, of which brachytherapy is an indispensable and important part (4).

At present, the brachytherapy techniques of cervical cancer are mainly divided into three categories intracavitary brachytherapy (ICBT), interstitial brachytherapy (ISBT), and intracavitary/interstitial brachytherapy (IC/IS-BT) (5). Although ICBT alone can achieve good curative effect on early cervical cancer, it has some limitations for locally advanced cervical cancer (6). ISBT can conform to the shape of the patients’ target area, but the dose of ISBT alone cannot cover the central area of the cervix, and it is more traumatic to the patient (7). Giving ISBT on the basis of ICBT, that is, IC/IS-BT can combine the advantages of both, which can not only ensure high-dose irradiation in the central area of the cervix, but also enable the dose to cover the target area conformally (8, 9). However, for cases with huge mass and severe parametrial invasion, even IC/IS-BT technology cannot achieve complete coverage of the target area. In addition, the operation of transplanting technology is more complicated and invasive. The incidence of bleeding complications is high, and it is easy to cause uterine and intestinal perforation. The implantation technique requires high doctor’s experience and equipment precision (10). For relatively underdeveloped areas, it cannot meet the requirements of human, material and financial resources, so it is difficult to carry out large-scale development. At the beginning of this century, some foreign scholars proposed ICBT synchronous external intensity modulated radiation therapy (IMRT), that is, after ICBT, the applicator was kept in the body and IMRT irradiation was performed immediately. For areas that cannot be covered by short-range irradiation, the maximum coverage of the target area can be achieved by supplementing with external irradiation IMRT (11). In recent years, many domestic and foreign scholars have theoretically carried out dosimetric feasibility analysis of internal and external fusion technology, and related research has gradually become a hot spot (12, 13). In this paper, the research progress of three-dimensional (3D) brachytherapy for cervical cancer in recent years is reviewed.

## 2 ICBT

ICBT is the use of an applicator to place a radioactive source inside the body’s cavities, such as the uterus and vagina. The earliest ICBT dates back to 1903, when Margaret-Cleaves cured 2 patients with cervical cancer with the radionuclide radium (14). After more than half a century of development, ^60^Co, ^137^Cs, and ^192^Ir have successively replaced radium, and ^192^Ir is currently the most widely used high-dose rate brachytherapy (HD-BT) (15). By far the most commonly used brachytherapy modality is still ICBT because of its simplicity and non-invasiveness to the patient. For patients with early-stage cervical cancer with small tumor volume and no parametrial invasion, the local control (LC) rate can reach 75% to 95% (16), but for patients with locally advanced cervical cancer, the LC is relatively low, about 45% to 80% (17). For patients with locally advanced cervical cancer, they are mainly characterized by large tumor size, irregular shape, eccentric location, and severe parametrial or other invasion (18). If only ICBT is irradiated, it produces a symmetrical pear-shaped dose distribution next to the uterine canal. In the case of limited organs at risk (OAR) doses, the target area cannot reach the curative dose, resulting in a high tumor recurrence rate (19). Yoshida et al. used the oval body model to calculate the high risk-clinical target volume (HR-CTV), and considered that ICBT was applicable when the HR-CTV volume was <18.8 cm^3^ (20). The study by Serban et al. compared the dosimetric differences between the ring intracavitary applicator and the Utrecht intraluminal applicator. It was found that no matter which applicator was used, the large target volume could not be covered by intracavitary radiotherapy alone (21). The above research results confirm that ICBT can achieve better therapeutic effect in the treatment of cervical cancer at an earlier stage, but it is not suitable for patients with locally advanced cervical cancer. In conclusion, ICBT technology is mainly suitable for cervical cancer in the early stage and small tumor size, or the position of the mass is symmetrically distributed relative to the uterine cavity and the parametrial tissue is not invaded in the three-dimensional brachytherapy.

## 3 ISBT

In view of the above-mentioned limitations of ICBT, the emergence of ISBT has brought new ideas of brachytherapy. ISBT is a method of transvaginal placement of a needle into the lesion and/or surrounding tissue using a perineal template or applicator. The main indications are: (1) huge mass; (2) involvement of the lower vaginal segment; (3) the development of the disease on one side or the inappropriate applicator for endoluminal therapy, such as cervical defect or vaginal fornix stenosis; (4) after subtotal hysterectomy; (5) vagina Stump cancer (22, 23). In the study of Ogorodniitchouk et al., 38 patients with locally advanced cervical cancer had a three-year overall survival (OS) rate of 81.6%, and both acute and late radiotherapy toxicity were within acceptable limits (24). In the study of Pinn-Bingham et al., the 5-year disease-free survival (DFS) and OS of 116 patients were 60% and 44%, respectively. 85.3% of patients achieved locoregional control (LC). Meanwhile, the 3-year disease-free survival (DFS) of patients with stage Ib, II, III, and IVa in this study were 59%, 67%, 71%, and 57%, respectively (25). The results suggest that ISBT can improve LC in patients with locally advanced cervical cancer. In the study of Bansal et al., the mean high-dose volume covered by 200% and 180% isodose surfaces was much larger in patients in the ICBT group than in the ISBT group (V200: 35.5 ± 1.1 vs 1 8.0 ± 4.0; V1 80: 42.2 ± 0.8 vs 39.1 ± 6.7; unit: cc) (26). ISBT still has deficiencies in target dose. Currently ongoing clinical studies on ISBT in cervical cancer are listed in **Table 1**.

**Table 1:** Ongoing clinical trials of ISBT in cervical cancer.

NCT identifier	Study phase	Treatment	Primary end point
NCT02957266	III	Radiation: Volumetric Arc Radiotherapy Radiation: ISBT Drug: Cisplatin Drug: Gemcitabine	Relapse Rate (local and/or distant) and Number of Deaths Due to Any Cause
NCT03249519	NA	Radiation: Radiation Radiation: ISBT Drug: Cisplatin Other: Hyperthermia	5 years OS
NCT04127435	NA	192Ir High Dose Rate ISBT	2 years LPFS
NCT03781271	NA	Device: Electromagnetic Navigation	Treatment Plan Target Volume and Organs at Risk Dosimetry
NCT03958357	NA	Device: AGA configuration	Performance of the AGA Venezia configuration applicator as assessed by the APQ

ISBT, interstitial brachytherapy; NA, Not Applicable; OS, overall survival; LPFS, local progression free survival; AGA, Advanced Gynecological Applicator; APQ, Applicator Performance Questionnaire.

ISBT is based on the tumor shape and boundary to reasonably distribute the needle, so that the dose is evenly distributed in the tumor area, and has a significant dosimetric advantage for locally advanced cervical cancer (27). Compared with ICBT, ISBT can significantly improve tumor target coverage, tumor local control rate and quality of life in patients with locally advanced cervical cancer (28). It should be emphasized that ISBT needs to be pre-planned before treatment, verified after implantation, and designed in a timely manner. It needs to have good quality control, including verification of radioactive source placement accuracy and dose accuracy. By sorting out relevant researches on ISBT at home and abroad, it is found that most foreign countries use transperineal planting templates, while domestically, templates are rarely used but freehand planting. In freehand ISBT, the implantation needle is highly flexible, and the depth and angle of the implantation needle will affect the treatment plan, which requires higher requirements for the operator, and the accuracy of needle insertion must be ensured, otherwise it may lead to massive bleeding and perforation (29, 30). Therefore, the implantation operation should be carried out under the guidance of images as much as possible to ensure the accurate and reliable needle insertion direction, position and depth. Liu et al. showed that CT-guided free-style implantation technique obtained satisfactory dose and volume histogram (DVH) parameters for patients with recurrent cervical cancer after radical resection and external beam radiotherapy, suggesting that ISBT can be applied to the treatment of patients with recurrent cervical cancer (30). However, it is impossible to achieve satisfactory results every time by inserting cloth needles with bare hands, and requires the operator to have a solid anatomical foundation and feel. In recent years, with the advancement of three-dimensional (3D) printing technology, 3D printing template-assisted implantation of needles has certain clinical application prospects (31). Compared with the implantation group, patients in the 3D printing template group could achieve higher target doses and lower doses of OARs (32). This technology can first design an individual needle track for cervical cancer patients, reduce the displacement error of the needle track, reduce the difficulty of operation, avoid multiple needle adjustments, reduce the risk of bleeding, and relieve the pain of the patient. However, each treatment needs to reprint the template according to the tumor regression of the patient, which increases the economic burden of the patient and takes a long time, which is difficult to be widely used in clinical practice.

## 4 IC/ISBT

In view of the above-mentioned deficiencies of ICBT or ISBT treatment, how to improve the effect of brachytherapy in patients with locally advanced cervical cancer and recurrence? Gaddis et al. reported the combined intracavitary implantation treatment in cervical cancer for the first time in 1983 (33). However, due to the limitations of the treatment conditions at that time, more patients (21.3%) suffered severe radiation reactions, so no follow-up research was carried out. In a prospective study in 2006, while achieving a 95% complete remission rate (21 patients), there were no serious early or persistent late post-radiotherapy adverse reactions (26). This confirms the efficacy of IC/ISBT. In the past 5 years, a number of studies have also demonstrated the advantages of IC/ISBT in terms of dosimetry and clinical efficacy (21, 34–37).

### 4.1 Indications

Currently, the generally accepted indications for IC/ISBT are (23, 38): ① large tumor size or poor regression after external irradiation; ② tumor location far from the uterine duct or obvious bias; ③ Irregular tumor shape; ④ Incomplete coverage of ICBT target volume; ⑤ Poor relative position between target volume and organ at risk; ⑥ Parametrium invasion; ⑦ patients with recurrence. The study of Yoshida et al. suggested that the dosimetric parameters of HR-CTV of different sizes were compared by intracavity, implantation, or a combination of the two. It was found that HR-CTV in the size of 4 cm × 3 cm × 3 cm was a critical value. Less than this volume is a better choice for ICBT, and when it is greater than this volume, IS or IC/ISBT is a better choice (20). Fokdal et al. found that the mean D90 of HR-CTV increased from 83 ± 14 Gy to 92 ± 13 Gy with IC/ISBT compared with ICBT alone. Patients with HR-CTV volume ≥30 cm^3^ had a 10% increase in 3-year LC. For HR-CTV < 30 cm^3^, no difference was found (39). For patients with recurrent cervical cancer, a study by Umezawa et al. (19) showed that 66.7% of patients who used ISBT achieved radiological and pathological complete remission (CR). The 2-year LC, progression-free survival (PFS) and OS of all patients were 51.3%, 20.0% and 60.8%, respectively (40). The results of the multiple studies mentioned above fully support the description of the indications for IC/ISBT at the beginning of this paragraph.

### 4.2 Development of source applicators

IC/IS brachytherapy uses hybrid applicators that originated from transperineal templates employed in ISBT, including the Syed-Neblett template and the Martinez Universal Perineal Intersttial Template (MUPIT) applicator (41, 42). However, these transperineal templates have limitations, including obstructing the field of view during the post-installation operation, limited freedom in needle placement, and a higher probability of deviation in implantation. To address these challenges, a feasible IC/IS hybrid applicator was produced by combining the advantages of intracavity and interstitial techniques. At present, the Utrecht-type (Utrecht) source applicator and the Vienna-type (Vienna, also known as Ring-type) source applicator are mainly used.

**Figure 1:**
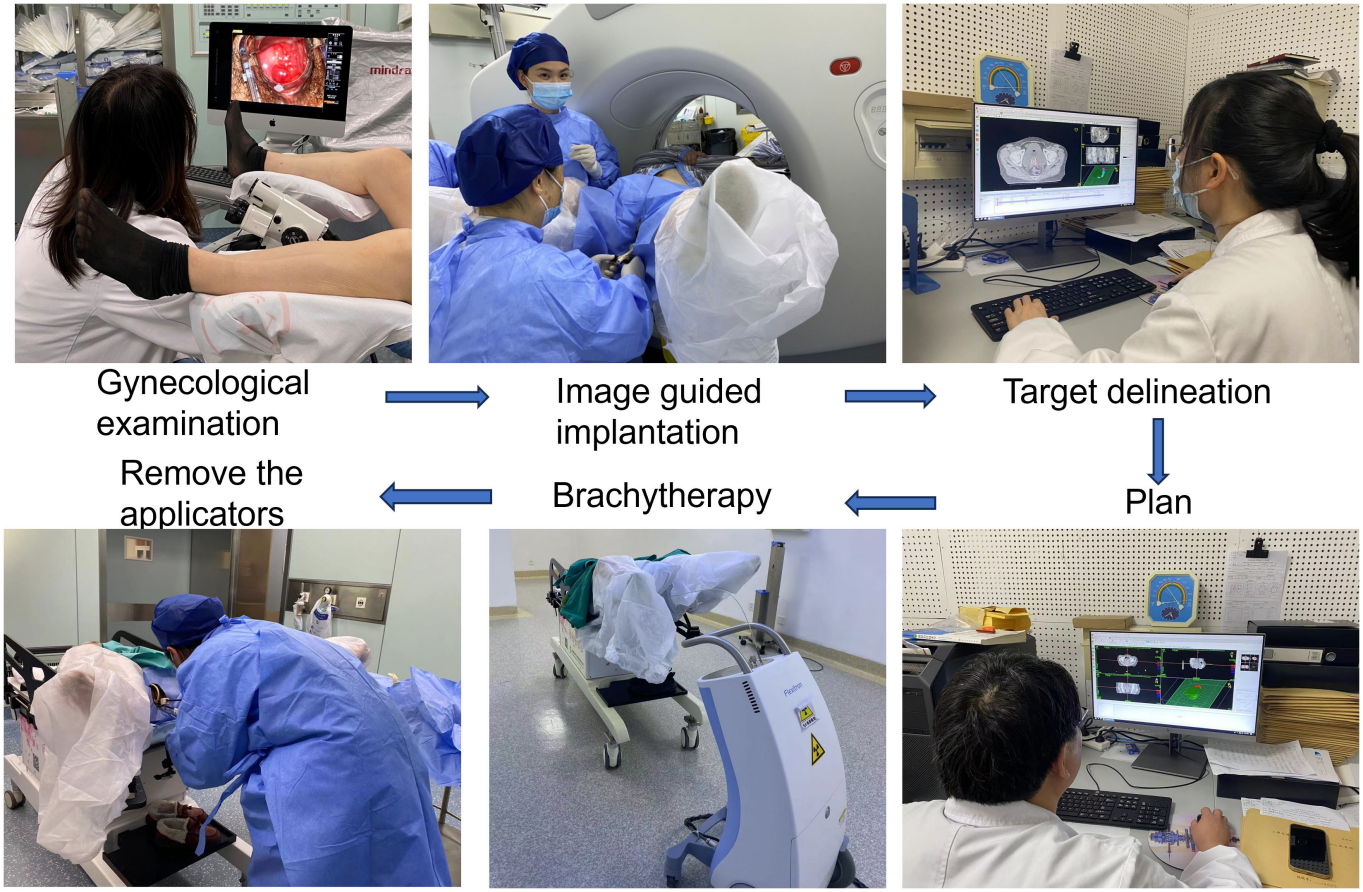
The flowchart of CT guided brachytherapy.

In recent years, the clinical research of other modes of hybrid applicators has further enriched the template of brachytherapy. For example, the Venezia applicator (consisting of an intraluminal series and two crescent-shaped ovals), allows for needle placement either parallel to the uterine canal or at an angle of up to 12° (43). Walter et al. confirmed that the clinical application of the Venezia applicator is feasible, significantly improving the dose coverage while sufficiently limiting the dose to organs at risk (44). At the same time, the combination of custom-made applicators and 3D printing technology has gained traction. To address the difficulties of applying traditional applicators to patients with vaginal stenosis and the challenge of guiding personalized needle trajectories, Lindegaard et al. introduced a self-made 3D printed vaginal template combined with traditional applicators. This approach, used for the treatment of stage IVa patients, simplified the procedure compared to the Ring type applicator (45). Kadoya et al. Also highlighted the benefits of 3D printing for individualized treatment, achieving high efficacy without serious side effects (46). While each medical center may adapt the choice of templates to the specific conditions of their patients, the Utrecht and Vienna applicators remain the most widely used. **Figure 1** presents the flowchart of CT-guided brachytherapy in our department.

### 4.3 Utilization of image-guided implant technology

The implantation methods guided by imaging technology mainly include X-ray, ultrasound, CT, and MRI. For the first time, Stock et al. conducted a detailed and specific study on the application of ultrasound guidance in posterior implantation. It is shown that the application of ultrasound can observe the target volume and normal tissue in real time and accurately place the needle during tumor interstitial implantation (47). The study of Weitmann et al. pointed out that the placement of the puncture needle under the guidance of ultrasound is a simple, non-radiation, safe and economical method. It established the status of ultrasound guidance in IC/ISBT (48).

For the imaging methods used in radiotherapy localization, CT and MRI are the most commonly used localization techniques. At the same time, the application of imaging technologies such as PET-CT in brachytherapy of cervical cancer provides us with more precise positioning of biological targets. When localized, CT over-displays the actual size of the lesion compared to MRI. Compared with CT, MRI positioning is more advantageous than CT in accurately controlling the scope of the target area and the dose limitation of organs at risk, and MRI is more accurate in the assessment of local invasion (49). There were no statistically significant differences in the mean HR-CTV volume and the median cumulative dose of OAR obtained by CT and MRI localization using the implantation technique. Regarding the 2-year OS data, the MRI group was better than the CT group (81% vs 56%, P = 0.036) (50). However, at present, most medical institutions at home and abroad do not have the conditions to use MRI for localization. In view of this situation, Mahantshetty et al. (27) adopted the method of transrectal ultrasound-assisted CT localization, which made the HR-CTV using CT localization more accurate, and obtained an effect equivalent to that of using MRI as the localization technology (39.1 ± 20 vs 39.0 ± 1 9; unit: cm^3^) (51).

In summary, image guidance is an important part of the application of brachytherapy, which can relatively intuitively present the target area and surrounding tissues. This is consistent with the research results of EMBRACE (52). In the absence of MRI localization, brachytherapy with CT localization is feasible. However, CT localization supplemented by real-time ultrasound may achieve a better therapeutic level.

### 4.4 Use of implant needles

**Figure 2:**
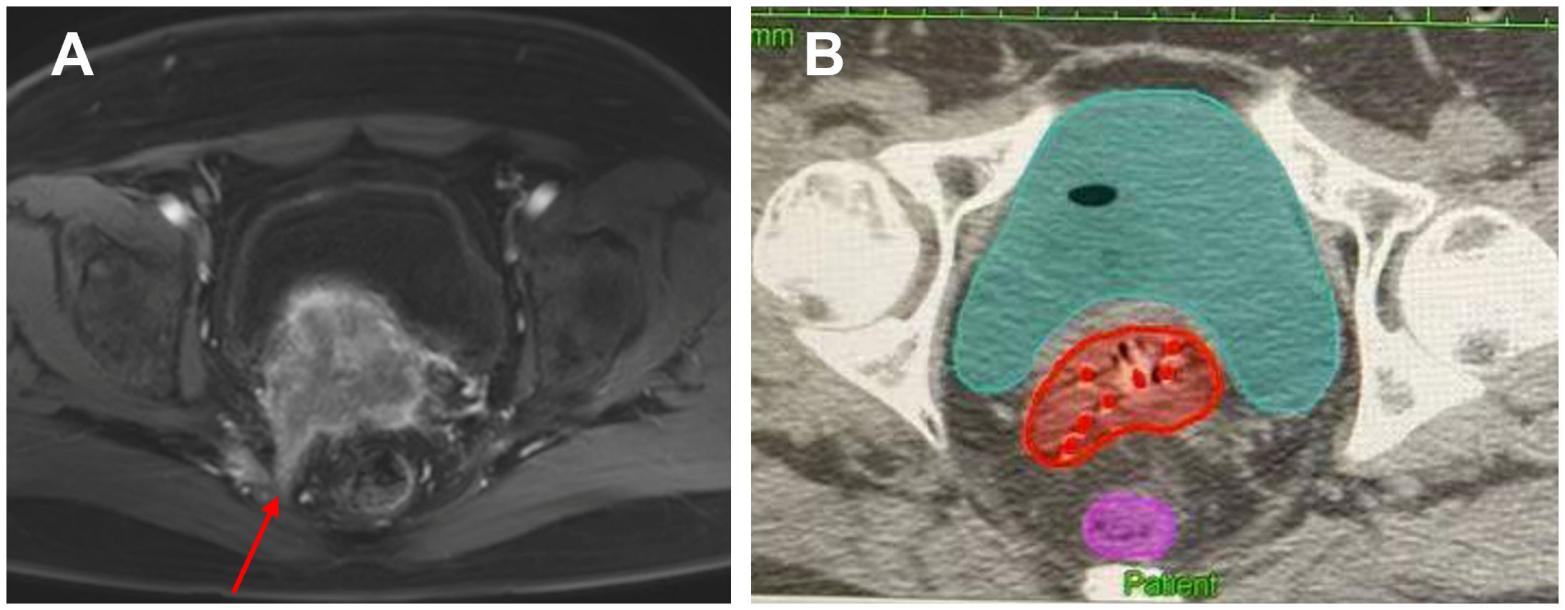
A cervical cancer patient with sacrococcygeal ligament invasion who was performed free handed implantation brachytherapy. **(A)** The red arrow indicates the invasion of the sacral uterine ligament before treatment. **(B)** The red dots in the picture represent the inserted needles, and the red area represents HR-CTV.

There is currently no clear guideline or consensus on the number and depth of implanted needles for reference. In the study of Kirisits et al., the needle tip should be located 5 mm above the suspected tumor location, because the 5 mm of the tip of the needle tip cannot be loaded with an effective radioactive source (53). At the same time, single insertion of 1 to 8 needles was used in this study, with an average of 3.5 needles (0 to 6 needles actually loaded, an average of 2.8 needles), and the average insertion depth was 18 mm. Sometimes fewer needles can achieve the desired target coverage (53). In the study by Fokdal et al., using three different pre-planning, the average number of planting needles was (5.3 ± 2.7), (5.3 ± 2.9) and (5. 4 ± 3. 0) roots, and the average planting depths were (33 ± 15) mm, (30 ± 10) mm, and (29 ± 11) mm, respectively (54). In a recent study, the effects of oval and ring-shaped applicators in combination with needles were compared. The average numbers of planting needles in the two groups were 3.5 and 4.8, respectively, and the average planting depth was 1.8 cm and 2.5 cm, respectively (21). According to the above research results, for locally advanced cervical cancer, the number of implanted needles for IC/ISBT is mainly concentrated in 1 to 6, and the implantation depth is concentrated in 20 to 40 mm. However, the angle of the implanting needle in the template is relatively fixed, so the free-style implanting needle still occupies an important position when the ideal coverage of the target area cannot be obtained. **Figure 2** shows an image of a cervical cancer patient with sacrococcygeal ligament invasion. We performed brachytherapy on the patient using free handed implantation technique. The use of the least number of needles in free hand implantation resulted in a satisfactory dose distribution.

### 4.5 Optimization of the treatment plan

For brachytherapy, planning optimization is essential. Different treatment planning optimization methods have their own advantages in calculating the residence time and residence position of the radioactive source for a given after loading catheter. Tanderup et al. pointed out the necessity of optimization. The optimization of the radiation source residence time significantly improved the uniformity, consistency, minimum target dose and high dose volume of the radiotherapy plan, while pointing out that the graphical optimization is better than the geometric optimization (55). The needle placement in virtual pre-planning can be reproduced in subsequent actual brachytherapy applications, resulting in significantly improved DVH parameters and rapid clinical needle placement (54). Compared with manual optimization, inverse planning simulated annealing (IPSA) significantly reduces the doses of the rectum and bladder without losing the target dose (56). Compared with volume-based optimization methods, IPSA significantly reduces the overall treatment planning time while reducing the dose of OAR (57). For the comparison of three optimization methods, forward optimization (FO), reverse optimization and hybrid inverse planning optimization (HIPO), recent clinical studies have shown that HIPO is only used in cervical cancer patients. A dosimetrically acceptable plan can only be produced with a larger number of needles inserted (58). It can be seen from the above that plan optimization is an indispensable link in brachytherapy, and a relatively high-quality treatment plan in terms of dosimetry can be obtained according to this method.

### 4.6 Clinical effects

Fokdal et al. compared the application of IC/ISBT and ICBT in patients with locally advanced cervical cancer through a multicenter study. IC/ISBT can increase the target dose, that is, the D90 of HR-CTV increased from 83 ± 14Gy to 92 ± 13Gy, while the dose of OAR did not change significantly. For patients with HR-CTV volume greater than 30 cm^3^, 3-year LC increased by 10%. Without increasing treatment-related late adverse reactions, local control of large tumors can be significantly improved, thereby improving the treatment rate of locally advanced cervical cancer (39). In recent years, related studies from other institutions have further confirmed the efficacy of IC/ISBT. In the clinical trial of Murakami et al., 52 people who met the experimental criteria were included in the follow-up, and the incidence of non-hematological adverse events of grade ≥ 3 was 1.9% (1/52) (59). Vízkeleti et al. included 21 patients. At a median follow-up of 11 months, the LC was 92.3% and the pelvic control rate was 86.5% (60). At the same time, no patients experienced grade 4 late adverse reactions. In another recent study by Murakami et al., the 2-year OS, PFS and LC of 42 patients were 81.6%, 54.4% and 80.2%, respectively. Local recurrence occurred in 7 patients (16.7%), and 3 patients had late adverse reactions of grade ≥ 3 (61). The above studies have shown the advantages of IC/ISBT: ① Dosimetry: The dose of OAR did not increase while increasing the target dose; ② Clinical effect: Better LC can be obtained in locally advanced cervical cancer, and few Patients with severe radiation toxicity.

## 5 Radioactive Seed Interstitial Brachytherapy

Radioactive seed implantation is more commonly used in patients with recurrent or metastatic cervical cancer. The interstitial implantation is permanent, and iodine 125 (^125^I) is currently the most commonly used radioactive source. Studies have shown that the therapeutic dose is clearly defined, and 130-150 Gy is an appropriate interval. At the same time, the consensus clarified that the indication for seed implantation is cervical cancer patients with recurrence after surgery or radiotherapy and chemotherapy (62). Related studies in the past 2 years have further confirmed the advantages of seed implantation in the treatment of patients with recurrent or metastatic cervical cancer. The results of Qua et al. suggested that the 1-year and 2-year local progression-free survival of 36 patients with recurrent pelvic cervical cancer after seed implantation were 34.9% and 20%, respectively. The local pain was significantly relieved with the survival benefit (63). In conclusion, seed implantation technology in the treatment of recurrent or metastatic cervical cancer, especially cervical cancer recurrence in the pelvis, reflects the advantages of safety and reliability, less trauma, significant local relief effect, and good pain relief effect.

## 6 ICBT + IMRT

ICBT combined with external IMRT radiotherapy, also known as internal and external fusion irradiation. That is, after the intracavity brachytherapy is completed, the applicator still temporarily resides in the patient. Immediately, image-guided supplemental external beam radiation therapy is performed on the linear accelerator with reference to the applicator and patient anatomy. This combined irradiation method of internal and external fusion is used to perform dose compensation for the target area that cannot be covered by ICBT. In the early 2000s, Low et al. and Wahab et al. introduced a new after-treatment approach for cervical cancer. They propose to guide IMRT external beam therapy through an applicator inside the patient (64, 65). That is, the source applicator is used as the marker point for positioning, the source applicator is used as the anatomical structure for field deployment during planning, and the patient’s position is calibrated with the patient’s body anatomical structure and the source applicator as the reference image during treatment. The target area that cannot be covered by intracavity irradiation is supplemented by external irradiation. On this basis, Duan et al. further proposed a method of combining high dose rate (HDR) brachytherapy with external beam intensity-modulated radiation therapy (IMRT) and analyzed its dosimetric feasibility. That is, the HDR intracavity brachytherapy plan is designed first, and then the underdose area of ​​the target area is supplemented by IMRT external irradiation. The internal and external fusion irradiation was compared with the traditional two-dimensional intracavitary irradiation (C-HDR) plan and the three-dimensional intracavitary irradiation plan (O-HDR). All 6 patients had V 95 greater than 95%. Although the O-HDR plan has a good target volume, the dose of adjacent organs at risk (OAR) is high. The C-HDR plan had the worst target wrapping, with V 95 less than 62% in 5 of 6 patients (66). Assenholt et al. selected 6 patients with locally advanced cervical cancer treated with IC/IS-BT, and simulated four planning methods: ICBT, IC/IS-BT, ICBT + IMRT, and IMRT alone. Dosimetric evaluation of these methods was performed separately. Comparative analysis found that the median target dose coverage was 74%, 95%, 96%, and 98% for ICBT, IC/IS-BT, ICBT + IMRT, and IMRT alone plans, respectively. Although the highest dose coverage was achieved by IMRT supplementation alone, the V60 of normal tissues and organs was 2 times that of several other planned modalities (67). Similar to Assenholt et al., Yin et al. found that the D 90, D 100 of HR-CTV and D 90, D 100, and V 100 of IR-CTV of ICBT + IMRT plan were higher than those of ICBT alone, and the D 2cc acceptance of OAR is lower than several other plans (13).

In conclusion, ICBT + IMRT technology is mainly applicable to the following three-dimensional brachytherapy of cervical cancer: (1) Patients with huge mass and severe parametrial invasion, who are unable or unsuitable for implantation; (2) For units that cannot meet the requirements of planting technology or planting conditions, it can be used as a supplement and alternative to IC/IS-BT technology.

## 7 Unsolved problems

Each radiotherapy center is not unfamiliar with the four treatment techniques of ICBT, IS-BT, IC/IS-BT and ICBT + IMRT in the three-dimensional brachytherapy of cervical cancer. The optimal clinical option is made according to the characteristics of the patients’ tumor and the conditions of the unit. But it is worth noting that, no matter which 3D brachytherapy technology is used, there are still some unsolved problems, which may affect the treatment effect. (1) Time and displacement factors. The 3D brachytherapy can only reach the treatment stage after the process of applicator placement, CT scanning, plan design, and plan evaluation. During this process, due to the patient’s transportation and waiting, the applicator will inevitably be displaced due to the time factor, resulting in the change of the position of the applicator having a greater impact on the dose distribution. It is recommended to fix the applicator inserted in the patient’s body as much as possible, and to avoid the patient waiting for a long time. In addition, in addition to the DVH map and isodose line, the weight of the dwell point needs to be considered during the planning evaluation to reduce the dose deviation caused by the change of the applicator position. (2) Positioning method factors. GEC-ESTRO recommends MRI for localization, and a localization scan, delineation of the target volume, and planning should be performed before each treatment. But at present, most radiotherapy centers in China cannot reach it. It is recommended to perform radiological examination before each treatment, and to delineate the target area and design the plan based on the results of the physical examination and CT localization images on the same day. (3) Factors of dose-fractionation mode. There is currently no specification of a clear standard dose splitting pattern. Therefore, some deviations in the therapeutic effect may also occur when different dose division methods are selected for treatment.

## 8 Outlook

The pursuit of precise treatment has always been the common goal of doctors and scholars in the field of brachytherapy. However, regardless of the improvement of hardware or software, the improvement of the most important “people” in brachytherapy is extremely lacking. The uncertainty of the operating doctor in the process of implanting the source applicator is difficult to quantify. No matter how high the accuracy of the radioactive source is in place, no matter how accurate the mathematical model may be, it may be meaningless because of the small changes in the doctor’s operation. Therefore, how to improve the doctor’s operation accuracy must be a problem that needs to be solved. The development of brachytherapy in the past was dosimetrically oriented, the study of how to make the radiation dose more accurate. At present, the development of brachytherapy needs to be guided by spatial positioning, and how to design the implantation path of the radioactive source more reasonably, and fully consider the soft tissue deformation during the implantation process and its corresponding optimization plan. At present, the 4D brachytherapy system with the addition of the time dimension has become a new milestone in adaptive brachytherapy (68, 69). Therefore, how to use computer deep learning and artificial intelligence to improve the target volume between fractionated treatments? Adaptability and treatment efficiency will become new challenges for the development of brachytherapy.

## 9 Conclusions

In this paper, four common techniques for 3D brachytherapy of cervical cancer were retrospectively summarized, and the dosimetric feasibility of internal and external fusion irradiation was also discussed. ICBT alone is suitable for patients with early-stage cervical cancer, but it is more limited for patients with locally advanced cervical cancer. ISBT alone that allows for conformal insertion based on target morphology, but results in under-volume in the central cervical region. IC/IS-BT has clear dosimetric advantages and efficacy in patients with locally advanced cervical cancer. However, the implantation technique is complicated and invasive, and the incidence of bleeding complications is high. External fusion irradiation has obvious dosimetric advantages for cervical cancer patients with large tumor volume and irregular geometry. And it does not need to be transplanted, the operation is simple, and it is non-invasive to the patient. However, the current internal and external fusion technology is mostly in the theoretical stage in the brachytherapy of cervical cancer. The number of clinical cases taken is relatively small, and there is no relevant report on routine clinical treatment. In the future, it is necessary to support the feasibility of internal and external fusion technology through a large number of clinical treatment data, in order to provide a new option for locally advanced cervical cancer after three-dimensional brachytherapy.
